# Input dependence of local field potential spectra: experiment vs theory

**DOI:** 10.1186/1471-2202-14-S1-P41

**Published:** 2013-07-08

**Authors:** F Barbieri, A Mazzoni, N K Logothetis, S Panzeri, N Brunel

**Affiliations:** 1 ISI Foundation, Turin, Italy; 2 Istituto Italiano di Tecnologia, Genoa, Italy; 3 Department of Psychology, University of Glasgow, UK; 4 Max Planck Institute for Biological Cybernetics, Tuebingen, Germany; 5 Departments of Statistics and Neurobiology, The University of Chicago, Chicago, USA

## 

How sensory stimuli are encoded in neuronal activity is a major challenge for understanding perception. A prominent effect of sensory stimulation is to elicit oscillations in EEG and Local Field Potential (LFP) recordings over a broad range of frequencies. Belitski et al. [1] recorded LFPs and spiking activity in the primary visual cortex of anaesthetized macaques presented with naturalistic movies and found that the power of the gamma and low-frequency bands of LFP carried largely independent information about visual stimuli, while the information carried by the spiking activity was largely redundant with that carried by the gamma-band LFPs.

To understand better how different frequency bands of the LFP are controlled by sensory input, we computed analytically the power spectrum of the LFP of a theoretical model of V1 (a network composed of two populations of neurons - excitatory and inhibitory), subjected to time-dependent external inputs modelling inputs from the LGN, as a function of the parameters characterizing single neurons, synaptic connectivity, as well as parameters characterizing the statistics of external inputs.

Our model consists in a two populations network of excitatory and inhibitory leaky integrate-and-fire neurons. Standard analytical methods using the Fokker-Planck formalism can be used to compute average firing rates of both populations in the asynchronous state of the network, as well as the region of parameters for which this state is stable ([2,3]). The power spectrum of the global activity and the LFP (sum of average excitatory and inhibitory currents onto pyramidal cells of the network) can also be computed in a network of finite size ([2]). Using linear response theory we then calculated the response of the network to a dynamic input ([4]). The final result was an equation giving the LFP power spectrum as a function of the intrinsic parameters of the network and of the parameters characterizing the dynamic input.

We then used the analytical expression of the LFP power to fit the experimental data of [1]. The data consists in LFP recordings from primary visual cortex of monkeys, during the presentation of a movie that last several minutes. In order to capture the LFP power changes during the movie presentation, the LFP traces were divided into 2 seconds non-overlapping scenes. We then fitted the LFP power of all the scenes with the same network parameters, but with input parameters free to vary scene-by-scene. We used a simplex method repeatedly applied for different initial conditions. The parameter set that was selected was the one that minimized the reduced chisquare, among sets for which the asynchronous state was stable.

The model provided excellent fits of the data. The fitting procedure permitted to extract the values of the firing rates of the excitatory and inhibitory populations and the parameters characterizing the external input for most of the scenes of the movie. These outcomes could be then correlated with experimental firing rates and the features of the movie itself, such as temporal and spatial contrast as well as orientation. We found a significant correlation both between firing rates extracted from fit and the multi-unit activity recorded during the movie and between the parameters characterizing the external input and the features of the movie.

These results show how an analytical approach can be used to estimate the key parameters underlying changes in the LFP spectral dynamics.
